# Detection and Genetic Analysis of Jingmen Tick Virus in Greece

**DOI:** 10.3390/microorganisms14102321

**Published:** 2026-10-12

**Authors:** Styliani Pappa, Katerina Tsioka, Smaragda Sotiraki, Danai Pervanidou, Anna Papa

**Affiliations:** 1 National Reference Center for Arboviruses and Haemorrhagic Fever Viruses, Department of Microbiology, Medical School, Faculty of Health Sciences, Aristotle University of Thessaloniki, 54124 Thessaloniki, Greece; s_pappa@hotmail.com (S.P.); aik.tsioka@gmail.com (K.T.); 2 Veterinary Research Institute, Hellenic Agricultural Organization (ELGO)—DIMITRA, Campus ELGO, Thermi, 57001 Thessaloniki, Greece; sotiraki@elgo.gr; 3 National Public Health Organisation (EODY), 15123 Athens, Greece; d.pervanidou@eody.gov.gr

**Keywords:** Greece, *Ixodes gibbosus*, Jingmen tick virus, phylogeny, *Rhipicephalus bursa*

## Abstract

Jingmen tick virus (JMTV) is an emerging, segmented RNA virus in the *Flaviviridae* family. Since its first identification in 2010 in China, JMTV has been detected in several parts of the world, primarily in ticks, but also in other arthropods, mammals, and even humans. The study included 400 adult ticks (333 *Rhipicephalus bursa*, 40 *Ixodes ricinus* and 27 *Ixodes gibbosus*) previously collected from livestock and the environment in various administrative regions of Greece. The ticks were grouped into 107 pools and screened for JMTV using an in-house real-time RT-PCR protocol. JMTV was detected in 41 (38.3%) tick pools (minimum infection rate 10.2%) in five of the seven regions tested. The virus positivity differed among tick species, hosts and regions. A single JMTV-positive *R. bursa* tick has been previously found co-infected with Aigai virus. Five positive pools with the highest viral load (Ct range 14.61–20.36) were subjected to metagenomic next-generation sequencing. Genetic analysis based on the complete coding sequences of all four RNA segments revealed that the sequences of the Greek strains cluster within JMTV Group 1, sharing 95.41% to 97.16% nucleotide identity with their closest relatives from Kosovo, Romania, and Türkiye. The results of the study confirm the wide distribution of JMTV in ticks in Greece, providing the first comprehensive genomic data for the virus in the country and highlighting the need for surveillance studies to assess any public health risk.

## 1. Introduction

According to the latest report of the International Committee on Taxonomy of Viruses, Jingmen tick virus (JMTV) belongs to the genus *Jingmenvirus* in the family *Flaviviridae*. The viruses of this group are segmented, with a positive-sense single-stranded RNA genome. Often described as flavivirus-like, they are genetically distinct; unlike classic flaviviruses, the JMTV genome consists of four segments (S), denoted as S1-S4. The S1, S2 and S3 segments are monocistronic, each containing a single open reading frame encoding one protein, while the S4 segment is bicistronic, with two overlapping open reading frames encoding two proteins [1]. S1 and S3 encode non-structural-like proteins, while S2 and S4 encode structural-like proteins. Specifically, S1 encodes a flavivirus-like RNA-dependent RNA polymerase (NSP1/flavivirus NS5-like protein); S2 encodes the envelope protein (VP1); S3 encodes a flavivirus-like helicase/protease (flavivirus NS2b-NS3-like complex protein); and S4 encodes the capsid protein (VP2) and the membrane protein (VP3). Segments S1 and S3 show clear flavivirus-related ancestry, while the other two appear to be unique to the JMTV [1].

JMTV was originally identified in 2010 in *Rhipicephalus microplus* ticks in the Jingmen region of Hubei Providence in China [1]. After the initial discovery, there were reports from additional regions of China [2,3] and other countries in Asia [4,5,6], as well as in Africa [7], America [8,9,10], and Europe [11,12,13,14]. Several viruses with similar genome structure have been included in the genus, such as Mogiana tick virus (MGTV) [10], Alongshan virus (ALSV) [15], Yanggou tick virus (YGTV) [16], Takachi virus (TAKV) [6], and Guaico culex virus (GCXV) [17].

JMTV has been detected in multiple tick species, such as *R. microplus*, *R. bursa, R. sanguineus* s.l., *Ixodes ricinus*, *I. sinensis*, *I. granulatus*, *Haemaphysalis longicornis*, *H. campanulate*, *H. flava*, and *Amblyomma javanense*; it has also been found in *Anopheles* and *Armigeres* mosquito species [18]. Besides arthropods, JMTV has been detected in a wide range of vertebrates, such as cattle, goats, sheep, camels, monkeys, rodents, and bats, indicating broad zoonotic potential [1,19,20]. Human infection has been documented, and symptoms related to acute JMTV infections include eschars at the tick-bite site, fever, fatigue, myalgia, headache, nausea, and vomiting. Febrile human cases following a tick bite have been reported in China, while JMTV RNA was co-detected in Crimean-Congo haemorrhagic fever (CCHF) cases in Kosovo [14]. A retrospective study among 206 CCHF-suspected patients hospitalized in Turkiye during the outbreak in 2022 showed that JMTV RNA was detected in 3 (1.5%) cases [21]. Furthermore, the virus was detected in human skin biopsies by in situ hybridization, and it was able to replicate in cell cultures, while IgG seroconversion has also been reported [2]. Given its potential pathogenicity in humans and efficient transmission by ticks, it was suggested that JMTV has significant public health implications [18].

JMTV group is divided into two distinct clades, the tick- and vertebrate- associated JMTV, and the insect-associated JMTV [19]. A recent review highlights the global distribution of arthropod- and vertebrate-associated JMTV strains, suggesting that their segmented genome structure and evolutionary links to flaviviruses are critical for elucidating virus evolution and host tropism [22]. Based on their geographic distribution, the tick- and vertebrate-associated JMTV strains are separated into two groups: Group 1, containing strains from Central America and Europe, and Group 2, containing strains from South America, Africa and Asia [4].

Metagenomic analyses in ticks from southwestern China (Guangxi region) uncovered multiple, near-complete JMTV sequences, including new variants, and provided evidence of recombination among genome segments, suggesting high virus genetic variation [23]. Furthermore, discovery of new JMTV-related viruses in ticks from wild animals in Brazil expanded the evolutionary breadth of this genus, suggesting high genetic diversity among tick-associated viruses [24].

Greece is a Mediterranean country that has a variety of ecosystems with exceptional biodiversity, providing optimal living conditions for many tick species. A recent review documented a total of 26 hard tick species and subspecies parasitizing domestic animals and humans [25]. Several studies in ticks identified viruses in the families *Nairoviridae*, *Flaviviridae*, and *Phenuiviridae* [26,27,28,29,30,31,32,33]. The aim of this study was to screen for JMTV in ticks collected in Greece, obtain whole genome sequences of the viral strains, and determine their genetic relationships with strains detected in other parts of the world.

## 2. Materials and Methods

### 2.1. Material

A total of 400 adult ticks (333 *Rhipicephalus bursa*, 40 *Ixodes ricinus* and 27 *Ixodes gibbosus*) were included in the study. They had been collected in 2024 across seven of the thirteen administrative regions of Greece, as part of the ongoing OH SURVector project funded by the European Commission (code 101132974). The ticks were collected from livestock (232 from goats, 164 from sheep, 1 from cattle and 3 from the environment). Ticks were identified to the developmental stage, genus and species level under a stereomicroscope; identification was based on morphological characteristics (e.g., scutum, capitulum, festoons, spiracular plates, and leg segmentation) according to standard taxonomic keys for Ixodid ticks [34,35,36,37]. Within that project, *R. bursa* ticks were tested individually for Crimean-Congo hemorrhagic fever virus (CCHFV) and Aigai virus (AIGV) [26], while the *Ixodes* spp. ticks were individually tested for tick-borne encephalitis virus (TBEV). In the present study, we used the remaining halves of the tick bodies, which had been stored at −80 °C. Among the 333 *R. bursa* ticks, two had been found AIGV-positive.

### 2.2. Nucleic Acid Extraction

Ticks were grouped into pools (1–5 frozen longitudinal halves of each tick per pool) according to species, origin (type of host or environment), date and site of collection. The tick pools were mechanically homogenized in phosphate-buffered saline with glass beads (diameter 150–212 μm) using the TissueLyser II cell disrupter (Qiagen, Hilden, Germany) at 40 Hz for 5 min to release the intracellular genetic material. Total nucleic acids were extracted from 200 μL of homogenate using the QIAamp cador Pathogen Mini Kit (Qiagen, Hilden, Germany) according to the manufacturer’s instructions. Genetic material was eluted in 45 μL of elution buffer and stored at −80 °C until further analysis. To prevent contamination and ensure safety, all tick specimens were processed according to standard biosafety protocols for potentially infected arthropods prior to molecular analysis.

### 2.3. Real-Time RT-PCR

Ticks were tested for JMTV RNA using an in-house real-time RT-PCR assay with primers (gTJ-seg1 F and gTJ-seg1 R) and probe (gTJ-seg1 P), designed to detect JMTV S2 [12]. The PCR reaction was performed using the SuperScript III One-Step RT-PCR system with Platinum Taq DNA polymerase (Thermo Fisher Scientific, Waltham, MA, USA) on a QuantStudio 5 Real-Time PCR system (Thermo Fisher Scientific). Five μL of RNA was added to a 20 µL reaction mixture consisting of 12.5 μL of 2x reaction mix, 0.5 μL of Taq Platinum enzyme, 800 nM of each primer, 400 nM of the probe, and PCR nuclease-free water. The cycling conditions were those described by the authors [12]. Samples with a cycle threshold (Ct) value less than 40 that demonstrated a distinct sigmoidal amplification curve were considered positive.

### 2.4. Estimation of Minimum Infection Rate

Assuming that only one tick in a positive pool was infected, the Minimum Infection Rate (MIR), expressed as a percentage, was calculated as the number of JMTV-positive tick pools divided by the total number of ticks tested, multiplied by 100.

### 2.5. Next-Generation Sequencing

Five tick pools with the lowest cycle threshold (Ct) values (indicating highest viral load) were selected for metagenomic next-generation sequencing (mNGS) on the Ion GeneStudio S5 System (Thermo Fisher Scientific, Waltham, MA, USA). First-strand cDNA synthesis was performed using SuperScript III Reverse Transcriptase (Thermo Fisher Scientific, Waltham, MA, USA) with 10 μL of total RNA and oligo(dT) primers, according to the manufacturer’s instructions. Subsequently, second-strand synthesis was carried out using Klenow Fragment DNA polymerase (New England Biolabs Inc., Ipswich, MA, USA). Libraries were prepared using the Ion 510, 520 & 530 Kit for Ion Chef (400 bp reads) and quantified with the Ion Library TaqMan Quantitation kit. The library was normalized to a final concentration of 30 pM and sequenced on an Ion Torrent S5 platform. The final single-end library was loaded onto an Ion 520 semiconductor sequencing chip following the manufacturer’s instructions. Raw sequencing reads were automatically processed for quality control, filtering, and adapter trimming within the Torrent Suite Software (version 5.18.1, Thermo Fisher Scientific, Waltham, MA, USA) utilizing default quality thresholds. The resulting high-quality reads were then imported into Geneious (version 7.1.3, Biomatters), where sequence assembly was executed via reference-based mapping against the JMTV isolate SY84 (GenBank Accession numbers NC_024113, NC_024112, NC_024114 and NC_024111 for S1, S2, S3 and S4, respectively), using the Map (refers to Geneious version 7.1.3) to reference workflow with medium sensitivity/default parameters.

### 2.6. Phylogenetic Analysis

The sequences of all four JMTV RNA segments from the five tick pools were compared against nucleotide sequence databases using the Basic Local Alignment Search Tool (BLASTn; https://blast.ncbi.nlm.nih.gov/, accessed on 15 November 2025) to determine statistical significance relative to their closest evolutionary relatives. The sequences were aligned with JMTV sequences retrieved from the GenBank Database using ClustalW 1.8.3. Phylogenetic trees were reconstructed based on the complete coding sequences of all four JMTV RNA segments using the Maximum Likelihood method with the best-fit nucleotide substitution model implemented in MEGA 12 software [38]. The robustness of the inferred tree topologies was assessed using 1000 bootstrap replicates.

## 3. Results

A total of 107 pools were formed from the 400 ticks in the study; 87 pools consisted of 333 *R. bursa*, 13 of 40 *I. ricinus* and 7 of 27 *I. gibbosus*. They had been removed from 232 goats, 164 sheep, one from cattle and three from the environment (Table 1). Most *R. bursa* ticks (205/333, 61.6%) were collected from goats, while regarding *Ixodes* spp. ticks, most *I. ricinus* (38/40, 95%) were collected from sheep, while all *I. gibbosus* were collected from goats (Table 1).

JMTV was detected in 41 (38.3%) tick pools (MIR 10.2%); specifically, the virus was detected in 39 of 87 (44.8%) *R. bursa* pools (MIR 11.7%) and 2 of 7 (28.6%) *I. gibbosus* pools (MIR 7.4%), while all 13 *I. ricinus* pools were negative. JMTV was detected in 45.6% and 29.8% of pools consisting of ticks removed from goats and sheep, respectively, and in one of the two pools with ticks collected from the environment; the respective MIRs were 11.2%, 8.5% and 33.3% (Table 1).

JMTV-positive pools were detected in five of the seven screened regions of Greece. The MIRs varied per tick species, origin and region. The MIRs per tick species and region are shown in Table 2.

The Ct values of the JMTV-positive tick pools in the real-time RT-PCR ranged from 14.71 to 38.64 (mean 29.9). The five pools selected for mNGS (those with the highest viral load) had Ct values ranging from 14.71 to 20.36. All five pools contained *R. bursa* ticks. Three pools contained ticks collected in West Greece, one pool contained ticks collected in Thrace (East Macedonia and Thrace region), while the fifth pool contained ticks collected from the environment in the region of Peloponnese, in the southern part of the country.

Full-length genome sequences were obtained from all five tick pools. Table 3 summarizes the characteristics and the assembly metrics of the mNGS analysis, including the total number of reads and the average sequencing depth for each genomic segment.

The BLAST analysis showed that the genetically closest JMTV sequences at the nucleotide level were those from Kosovo, Romania and Türkiye, with identities ranging from 95.41% to 97.16%. (Table 4).

Two of the five pools tested by mNGS (WestGreece_2 and WestGreece_3) contained ticks in which AIGV had been previously detected. The WestGreece_2 sample contained only one *R. bursa* tick collected from sheep, which was co-infected with AIGV. The WestGreece_3 pool contained four *R. bursa* ticks, also collected from sheep, of which one was AIGV-positive; when the four ticks were tested individually for JMTV, the virus was detected in three ticks that were AIGV-negative, while it was not detected in the AIGV-positive tick. Therefore, JMTV and AIGV co-infection was detected in one *R. bursa* tick.

**Figure 1:**
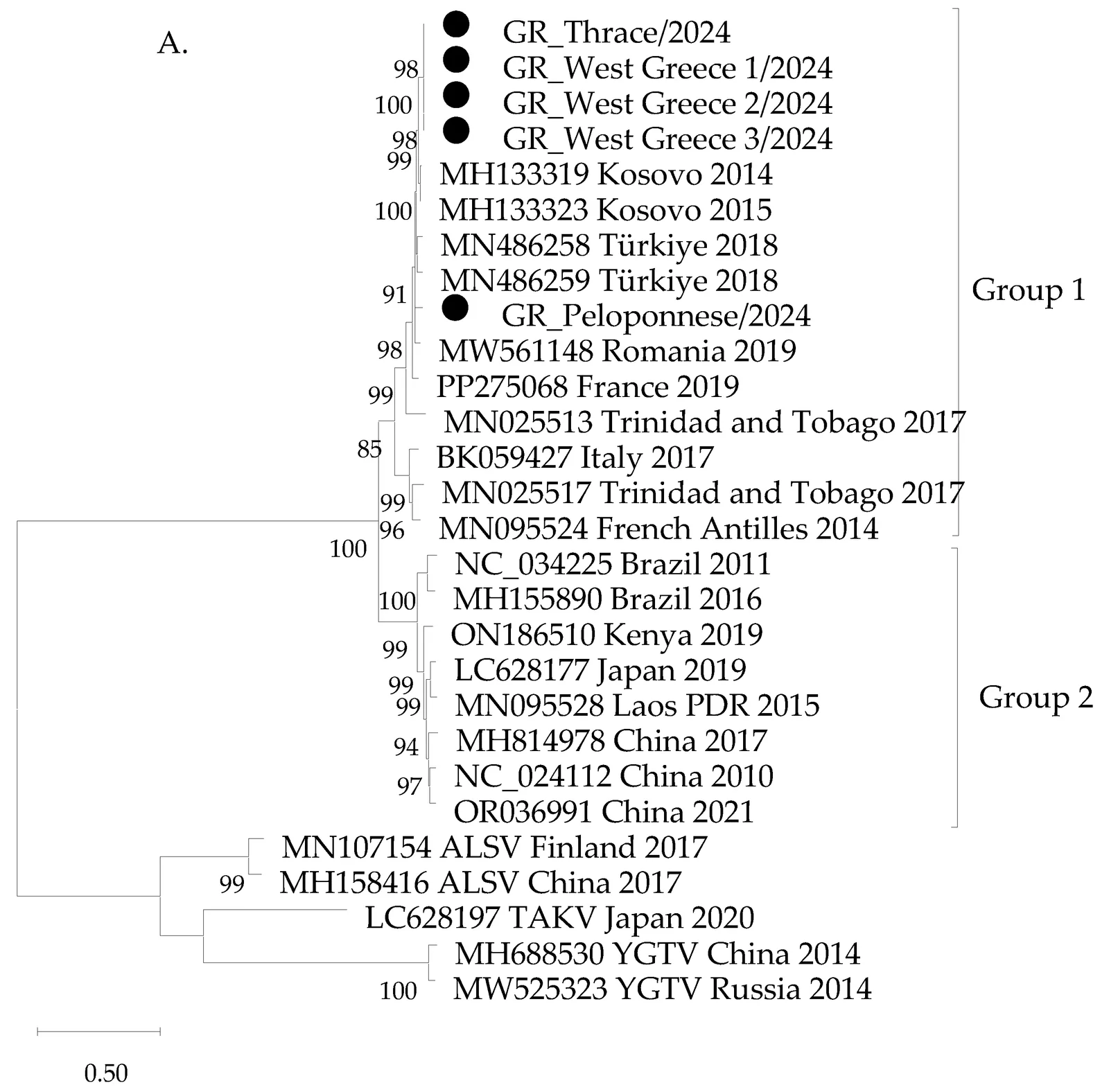

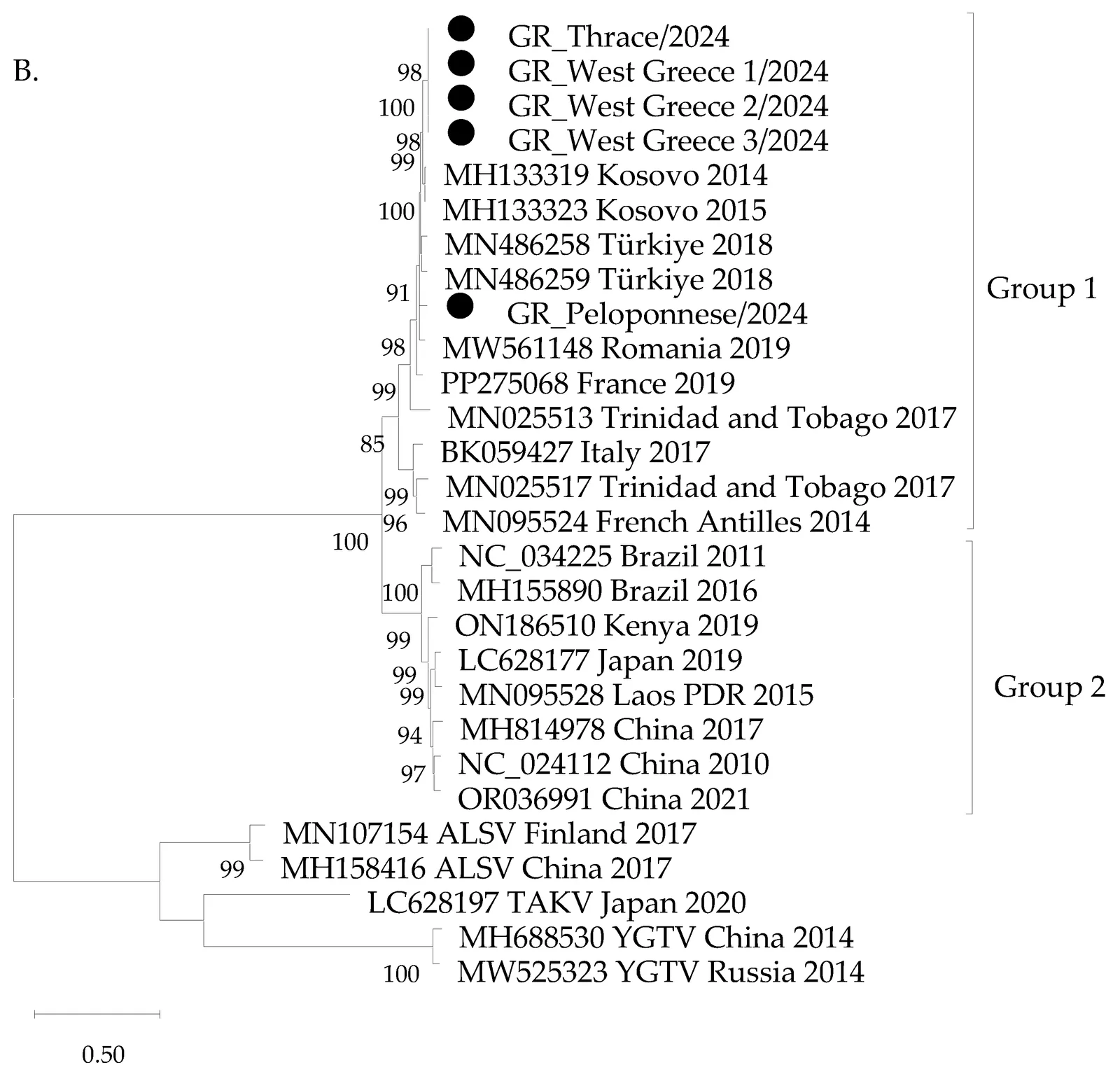

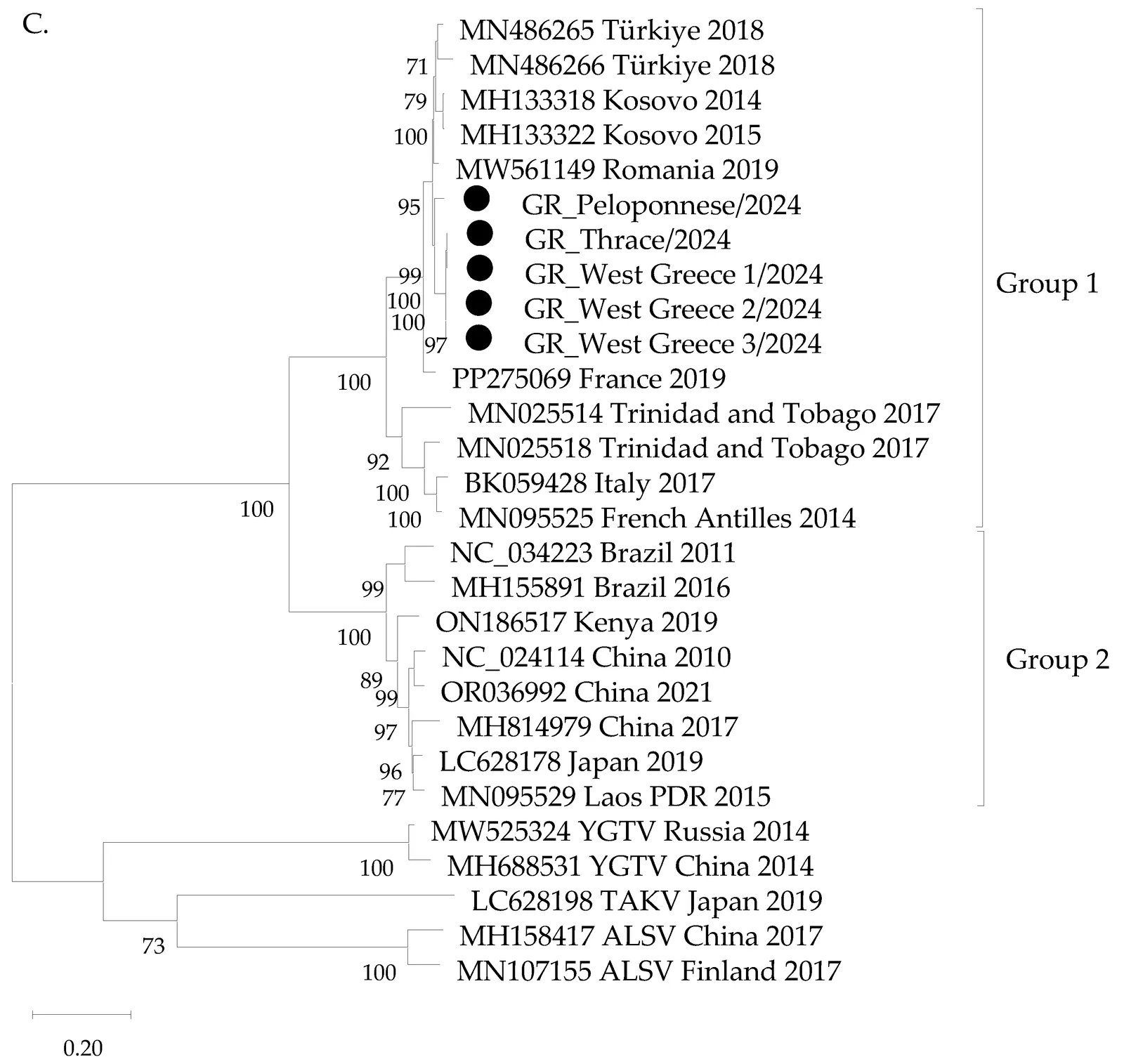

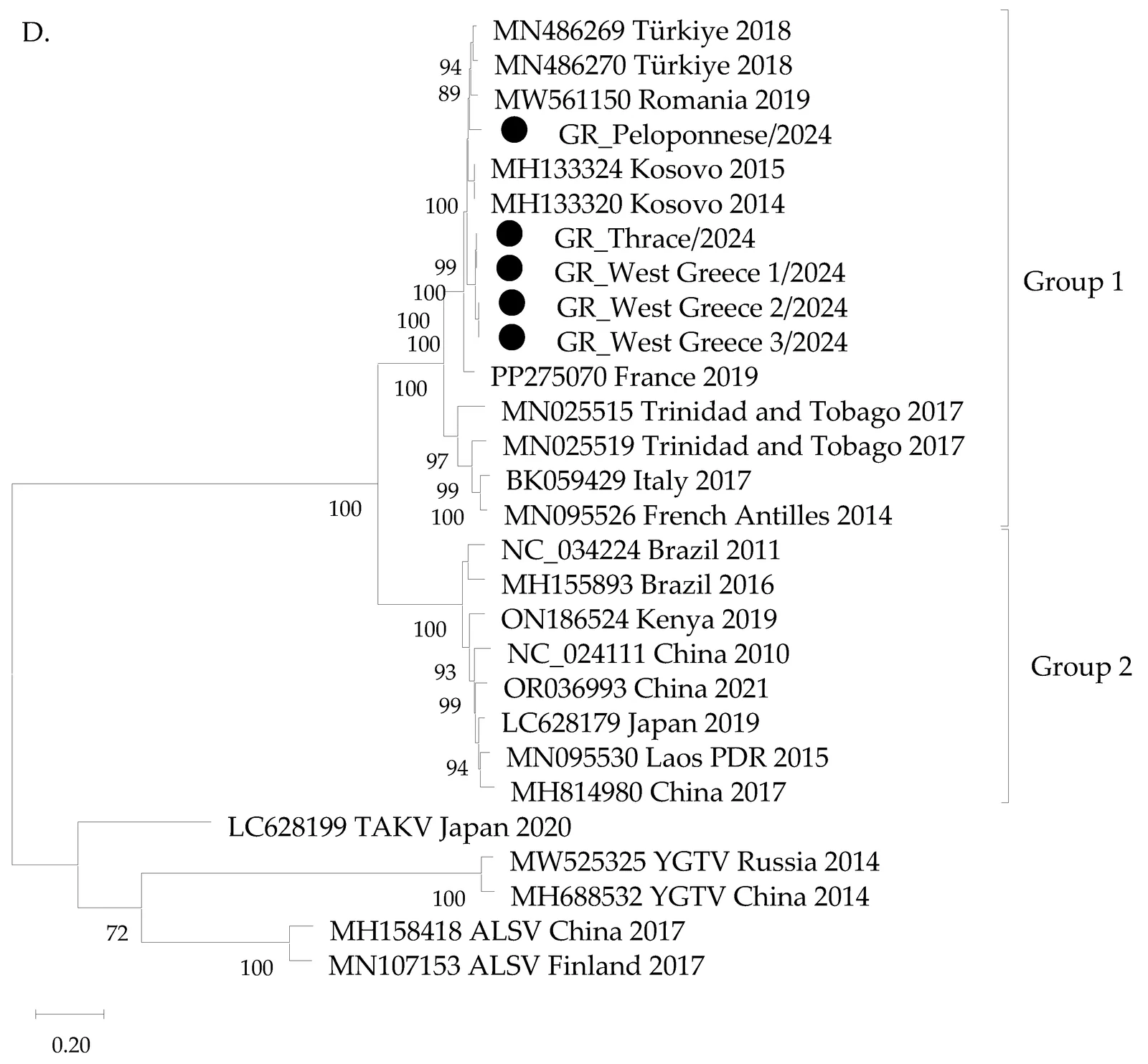
Maximum likelihood phylogenetic trees based on the complete coding sequences of JMTV RNA segments: (**A**): segment 1; (**B**): segment 2; (**C**): segment 3; and (**D**): segment 4. The trees were constructed with 1000 replicates; bootstrap values >70% are shown. Phylogenetic analysis of JMTV using complete coding sequences from this study is indicated with black circles.

Phylogenetic analysis of the complete coding sequences of the JMTV RNA segments placed all five Greek sequences within Group 1, together with strains from Europe and Türkiye (Figure 1A–D). Trees were inferred using the Maximum Likelihood method under the General Time Reversible model with 1000 bootstrap replicates. The initial tree was selected based on the highest log-likelihood among Neighbor-Joining and Maximum Parsimony trees. Evolutionary rate variation among sites was modeled using a discrete Gamma distribution with five categories.

Slight differences can be seen in the phylogeny of the four segments. While the Greek sequences form a unique subclade in S1, GR_Peloponnese/2024 clusters slightly differently in S3, and especially in S2 and S4. This resulted in higher nucleotide differences with the other Greek strains. Specifically, the nucleotide differences among the Greek sequences were 0–3.6% in S1, 0–5.5% in S2, 0–4.2% in S3, and 0–6.4% in S4. The greatest differences were observed in the pool GR_Peloponnese/2024, which contained two *R. bursa* ticks collected from the environment in the Peloponnese region.

## 4. Discussion

JMTV is a recently identified tick-borne flavivirus with zoonotic potential. It has been detected in several parts of the world, primarily in ticks, but also in other arthropods, mammals, and even in humans. The aim of this study was to find out whether JMTV is present in Greece. Since the virus has often been detected in *Rhipicephalus* spp. (mainly) and *I. ricinus* ticks, we included in the study *R. bursa*, *I. ricinus* and *I. gibbosus* ticks that had been collected previously as part of another project.

We found that JMTV is widely distributed in the country, as it was detected in five of the seven screened administrative regions of Greece. Although the prevalence of JMTV could not be determined precisely due to pooled rather than individual tick testing, the data indicate a high MIR (10.2%). Specifically, JMTV was detected in 38.3% of the tested tick pools, like the rates found in studies conducted in Kenya (45.8%), China (53–63%), Brazil (25–67%) and other countries [18,39,40]. This finding suggests that JMTV is widely established within the tick populations across Greece, resulting in an increased risk of JMTV transmission among ticks through co-feeding in animals [4]. It cannot be excluded that the virus detected in the ticks collected from the animals might originate from the animal blood. However, it was also found in the pool of two *R. bursa* ticks collected from the environment in the Peloponnese Region. This specific strain (GR_Peloponnese/2024) was the one with the greatest genetic difference from the other strains; it is not known whether this difference is related to the origin of the tick or the location.

Among the three tick species tested, JMTV was detected in 44.8% of *R. bursa* pools with MIR 11.7%, and 28.6% of *I. gibbosus* pools with MIR 7.4%, while the 13 *I. ricinus* pools were negative. To our knowledge, this is the first detection of JMTV in *I. gibbosus* ticks. *I. gibbosus* is prevalent in the central and eastern Mediterranean Basin and prefers drier and warmer ecosystems than those preferred by *I. ricinus* [41]. Since the number of *Ixodes* spp. ticks tested was relatively low, the statistical power for comparison of JMTV positivity among species is limited.

JMTV detection in both *R. bursa* and *I. gibbosus* in this study is in line with numerous studies worldwide, which suggests that the virus has a wide vector and host adaptation [18]. This means that JMTV has evolved in a complex way because of interactions between a wide variety of tick and host species, thus undergoing heavy selective pressures [40]. The fact that most Ixodid ticks require two or three different hosts to complete their life cycle from larva to nymph and adult (two- or three-host ticks like *R. bursa* and *I. gibbosus*, respectively) interferes with virus evolution by imposing strict genetic bottlenecks, forcing the virus to adapt to drastically different physiologies.

The high detection rate of JMTV in *R. bursa* and *I. gibbosus* in Greece suggests that these ticks might play an important role in the local ecology of the virus. It is known that the detection of a pathogen in ticks does not mean that this tick species is a competent vector of this specific pathogen, since vector competence refers to the genetic capacity of a tick not only to acquire and maintain a pathogen but also to transmit it efficiently [42]. Studies have shown that different pathogens may follow similar strategies (e.g., manipulation of the immune response) to infect vectors and facilitate the multiplication and transmission, while some pathogens use these strategies to infect both tick and mammalian hosts [42]. Tick studies are needed to identify the competent vector(s) of JMTV.

All whole-genome JMTV sequences of the study cluster within Group 1, which is defined as the Central American and European Group [4]. BLAST analysis of all four segments revealed high nucleotide identity (95.41–97.16%) with viral sequences reported from Kosovo (MH133317, MH133323, MH133324), Romania (MW561149), and Türkiye (MN486270) [5,14,43]. The similar clustering of the Greek sequences in Group 1 suggests a common evolutionary history and a potential shared mechanism of dispersal, providing evidence of a common evolutionary clade circulating within Southeastern Europe and the Mediterranean basin. Since Greece is at the meeting point of three continents (Asia, Africa and Europe), with numerous stopping sites of migratory birds, new virus introductions and recombination or reassortment events can be expected. Specifically, reassortment occurs when different viral strains co-infect the same host (tick or animal) and exchange entire genome segments between strains of the same virus species or different virus species/subtypes; these events are common in tick-borne segmented viruses [44]. Genetic recombination and reassortment have been reported in Jingmen viruses, which not only complicate their evolutionary relationships but also raise the risk of these viruses to humans [4].

Regarding geographic distribution, a recent study reported a strong correlation between JMTV genetic distances and latitude, supporting a latitude-dependent evolutionary pattern [45]. This finding explains the similar clustering of strains from Trinidad and Tobago, Türkiye, Kosovo, and Romania, with the addition of strains from Greece (Figure 1A–D).

Currently, JMTV has been detected on four continents (Asia, Africa, America, and Europe) [18]. However, its pathogenicity has not been elucidated yet. There are few reports of JMTV in humans, presenting mild febrile illness after a tick bite, and there is evidence of potential pathogenicity due to its ability to replicate in human cell cultures and to induce seroconversion [2]. Therefore, more studies are needed to gain a better insight into the spectrum of symptoms JMTV can cause in humans and the mechanisms of its pathogenesis. It is advisable to include JMTV in the differential diagnosis of tick-borne clinical cases.

Viral metagenomics and screening studies of clinical cases in Kosovo have detected JMTV RNA in clinical CCHF cases [14], while a study in Turkiye reported JMTV isolation following inoculation of a CCHFV-positive tick pool [5]. It is not clear whether JMTV can contribute somehow to the course and/or the outcome of CCHFV infection [21]. This knowledge is very important, as the up-to-date results show that JMTV has already been detected in CCHF endemic countries (e.g., Turkiye, Kosovo, China). In the present study, JMTV was detected in a single *R. bursa* tick co-infected with AIGV, an orthonairovirus which until 2021 was classified as CCHFV genotype VI [46]; its prototype strain, AP-92, was originally isolated from *R. bursa* ticks collected from a goat in Vergina village (ancient Aigai) in northern Greece [47]. Clinical studies, especially those combined with mNGS, could identify the role of JMTV as a co-infecting microorganism. Furthermore, serological surveys in livestock and human populations could also be of added value to determine the actual exposure rate, zoonotic impact, transmission dynamics, and clinical significance of JMTV.

## 5. Conclusions

This study reports the detection and wide geographical distribution of JMTV in *R. bursa* and *I. gibbosus* ticks in Greece, with MIRs differing among tick species, hosts and regions. These results extend the known geographical range of the virus, offering insights into JMTV epidemiology. The genetic characterization of the Greek viral strains based on full-length genomes showed that all sequences cluster into JMTV Group 1, together with sequences from Kosovo, Romania, and Türkiye, providing essential genomic data for the genetic diversity and evolutionary dynamics of the virus. The detection of JMTV in a single *R. bursa* tick, previously found positive for AIGV, highlights the complex, interconnected nature of tick-borne pathogens in livestock, warranting further investigation. Since recent studies showed that JMTV has zoonotic potential, there is a need for integrated surveillance strategies and studies to better understand its role in public health.

## Figures and Tables

**Table 1:** Number of ticks, pools, JMTV-positive pools and MIRs per tick species and origin (host/environment).

Origin	*Rhipicephalus bursa*		*Ixodes ricinus*		*Ixodes gibbosus*		Total	
	Ticks (Pools)	Positive Pools (MIR %)	Ticks (Pools)	Positive Pools (MIR %)	Ticks (Pools)	Positive Pools (MIR %)	Ticks (Pools)	Positive Pools (MIR %)
Goat	205 (50)	24 (11.7)	0 (0)	0 (0)	27 (7)	2 (7.4)	232 (57)	26 (11.2)
Sheep	126 (36)	14 (11.1)	38 (11)	0 (0)	0 (0)	0 (0)	164 (47)	14 (8.5)
Cattle	0 (0)	0 (0)	1 (1)	0 (0)	0 (0)	0 (0)	1 (1)	0 (0)
Environment	2 (1)	1 (50.0)	1 (1)	0 (0)	0 (0)	0 (0)	3 (2)	1 (33.3)
Total	333 (87)	39 (11.7)	40 (13)	0 (0)	27 (7)	2 (7.4)	400 (107)	41 (10.2)

**Table 2:** Number of ticks, tick pools, JMTV-positive pools and MIRs per tick species and region of Greece.

	*Rhipicephalus bursa*		*Ixodes ricinus*		*Ixodes gibbosus*		Total	
Region	Ticks (Pools)	Positive Pools (MIR %)	Ticks (Pools)	Positive Pools (MIR %)	Ticks (Pools)	Positive Pools (MIR %)	Ticks (Pools)	Positive Pools (MIR%)
East Macedonia and Thrace	58 (13)	4 (6.9)	0 (0)	0 (0)	27 (7)	2 (7.4)	85 (20)	6 (7.0)
Central Macedonia	25 (7)	5 (20.0)	21 (6)	0 (0)	0 (0)	0 (0)	46 (13)	6 (13.0)
West Macedonia	60 (18)	0 (0)	18 (6)	0 (0)	0 (0)	0 (0)	78 (24)	0 (0)
Central Greece	21 (6)	4 (19.0)	0 (0)	0 (0)	0 (0)	0 (0)	21 (6)	5 (23.8)
Epirus	11 (4)	0 (0)	1 (1)	0 (0)	0 (0)	0 (0)	12 (5)	0 (0)
West Greece	126 (30)	21 (16.7)	0 (0)	0 (0)	0 (0)	0 (0)	126 (30)	21 (16.7)
Peloponnese	32 (9)	5 (15.6)	0 (0)	0 (0)	0 (0)	0 (0)	32 (9)	5 (15.6)
Total	333 (87)	39 (11.7)	40 (13)	0 (0)	27 (7)	2 (7.4)	400 (107)	41 (10.2)

**Table 3:** Characteristics and assembly data of the five JMTV-positive *R. bursa* tick pools tested by mNGS.

Pool ID	No of Ticks per Pool	Origin	Ct	Region	Total Data (MB)	Total Reads	Mean Length (bp)	JMTV Specific Reads	Average Depth	Coverage S1, S2, S3, S4 (%)
GR_Peloponnese/2024	2	Environment	20.36	Peloponnese	58.8	193,430	304	3346	66.25x	99.2, 100, 94.7, 99.8
GR_WestGreece-1/2024	4	Goat	22.97	West Greece	33.8	111,553	302	71,651	5753.25x	100, 100, 99.1, 100
GR_WestGreece-2/2024	1	Sheep	18.35	West Greece	73.0	257,783	283	7811	407.75x	100, 100, 100, 100
GR_WestGreece-3/2024	4	Sheep	24.66	West Greece	138.2	431,934	320	12,734	1288.00x	100, 100, 99.5, 100
GR_Thrace/2024	4	Goat	14.71	East Macedonia and Thrace	53.3	175,181	304	167,874	7482.25x	100, 100, 99.8, 100

**Table 4:** Nucleotide identity of JMTV segment sequences to their genetically closest viral strains.

Segment (Pool ID)	Closest Relative Sequence (Ac. No)	Country	Identity nt (%)
S1 (all pools)	MH133317	Kosovo	96.32–96.52
S2 (all pools)	MH133323	Kosovo	95.82–97.13
S3 (all pools)	MW561149	Romania	96.95–97.16
S4 (WG-2, WG-3)	MH133324	Kosovo	95.50
S4 (WG-1, EMT, PEL)	MN486270	Türkiye	95.41–95.78

## Data Availability

The sequencing read data generated in this study have been deposited in the European Nucleotide Archive (ENA) under project accession number PRJEB122588.
